# Maternal Exposure to Polychlorinated Biphenyls and Asthma, Allergic Rhinitis and Atopic Dermatitis in the Offspring: The Environmental Health Fund Birth Cohort

**DOI:** 10.3389/fphar.2022.802974

**Published:** 2022-04-06

**Authors:** Maya Berlin, Hadar Flor-Hirsch, Elkana Kohn, Anna Brik, Rimona Keidar, Ayelet Livne, Ronella Marom, Amit Ovental, Dror Mandel, Ronit Lubetzky, Pam Factor-Litvak, Josef Tovbin, Moshe Betser, Miki Moskovich, Ariela Hazan, Malka Britzi, Itai Gueta, Matitiahu Berkovitch, Ilan Matok, Uri Hamiel

**Affiliations:** ^1^ Clinical Pharmacology and Toxicology Unit, Shamir Medical Center (Assaf Harofeh), Affiliated to Sackler Faculty of Medicine, Tel-Aviv University, Tel Aviv, Israel; ^2^ Division of Clinical Pharmacy, Institute for Drug Research, School of Pharmacy, Faculty of Medicine, Hebrew University of Jerusalem, Jerusalem, Israel; ^3^ Department of Neonatology, Shamir Medical Center (Assaf Harofeh), Affiliated to Sackler Faculty of Medicine, Tel-Aviv University, Tel Aviv, Israel; ^4^ Departments of Neonatology and Pediatrics, Dana Dwek Children’s Hospital, Tel Aviv Medical Center, Affiliated to Sackler Faculty of Medicine, Tel-Aviv University, Tel Aviv, Israel; ^5^ Department of Epidemiology, Mailman School of Public Health, Columbia University, New York, NY, United States; ^6^ Division of Obstetrics and Gynecology, Shamir Medical Center (Assaf Harofeh), Affiliated to Sackler Faculty of Medicine, Tel-Aviv University, Tel Aviv, Israel; ^7^ Residues Lab, Kimron Veterinary Institute, Beit-Dagan, Israel; ^8^ The Institute of Clinical Pharmacology and Toxicology, Department of Medicine, Sheba Medical Center, Affiliated to Sackler Faculty of Medicine, Tel-Aviv University, Tel Aviv, Israel; ^9^ Department of Pediatrics, Shamir Medical Center (Assaf Harofeh), Zerifin, Affiliated to Sackler Faculty of Medicine, Tel-Aviv University, Tel Aviv, Israel

**Keywords:** polychlorinated biphenyls (PCBs), endocrine-disrupting chemicals (EDCs), allergy, asthma, atopic dermatitis, pregnancy, allergic rhinitis

## Abstract

**Background:** Polychlorinated biphenyls (PCBs) are persistent organic pollutants banned for use worldwide. Due to their biodegradation resistance, they accumulate along the food chain and in the environment. Maternal exposure to PCBs may affect the fetus and the infant. PCBs are immunotoxic and may damage the developing immune system. PCBs are associated with elevated IgE antibodies in cord blood and are considered to be predictive of atopic reactions. Several studies on the association between prenatal exposure to PCBs and atopic reactions were previously published, albeit with conflicting results.

**Objectives:** To examine the association between maternal PCBs levels and atopic reactions in their offspring.

**Methods:** During the years 2013–2015, a prospective birth cohort was recruited at the delivery rooms of Shamir Medical Center (Assaf Harofeh) and “Dana Dwek” Children’s Hospital. Four PCBs congeners were investigated: PCBs 118, 138, 153, and 180. In 2019, when children reached the age of 4–6 years, mothers were interviewed using the ISAAC questionnaire to assess symptoms of atopic reactions, including asthma, allergic rhinitis, and atopic dermatitis.

**Results:** One hundred and fifty mother-child dyads were analyzed. No significant differences were found in the median serum PCBs concentrations of each studied congener or total PCBs for asthma, allergic rhinitis, atopic dermatitis diagnosis, or parent-reported symptoms. No association was found between exposure to total PCBs and the risk for asthma symptoms or diagnosis, adjusted to maternal age and family member with atopic condition: aOR = 0.94, 95%CI: (0.88; 0.99). No association was observed between each studied PCB congener and asthma symptoms or diagnosis. The same results were found also for other studied outcomes—allergic rhinitis and atopic dermatitis.

**Conclusion:** Our study joins a series of previous studies that attempt to shed light on environmental exposures *in utero* as influencing factors for atopic conditions in children. Our results reflect the complexity of the pathophysiology of these phenomena. No relationship between maternal serum PCBs levels was demonstrated for asthma, allergic rhinitis, or atopic dermatitis. However, additional multi-participant studies, with longer, spanning into later pediatric age follow up are needed.

## Introduction

Atopic conditions are complex traits, most probably caused by an interaction of multiple disease susceptibility genes and environmental factors (Sengler et al., 2002). During the last century prevalence of atopic conditions has been on the rise. Numerous studies link exposure to various domestic and industrial environmental pollutants with bronchial wheezing and atopic morbidity (Masoli et al., 2004; Pope and Dockery, 2006; Clark et al., 2010; WHO Regional Office for Europe, 2013). Several studies in Israel have found an association between exposure to such environmental pollutants and childhood asthma and respiratory morbidity (Portnov et al., 2012; Greenberg et al., 2015; Greenberg et al., 2019).

Polychlorinated biphenyls (PCBs) are a large family of persistent organic pollutants, industrial chemicals, once used widely as non-flammable lubricants and insulators. This family of PCBs is considered as one of the most dominant pollutants worldwide (Agency for Toxic Substances and Disease Registry, 2000; Bergman et al., 2012). PCBs were banned from production in late 1970 in the United States and from 2001 worldwide by the Stockholm Convention on Persistent Organic Pollutants (United Nations Environment Programme, 2018). However, their chemical properties, such as a long half-life and fat solubility, cause them to be preserved in soil, water, and food chain, and consequently in human tissues (Domingo, 2012). PCBs are readily absorbed from the environment into the food chain, rendering human and animal exposure ubiquitous. Human exposure is mainly through consuming fatty foods like fish, meat, and dairy products (Freels et al., 2007; Fernández-González et al., 2015). PCBs 118, 138, 153, 180 are among the most frequently detected congeners in human adipose tissue and represent long-lasting exposure (Bonefeld-Jørgensen et al., 2001; Freels et al., 2007; Müllerová and Kopecký, 2007; Domingo, 2012). PCBs 138, 153, and 180 have the highest detection frequencies in the US population and contributed to 80% of the total PCBs in human serum (Faroon and Ruiz, 2015).

Several studies have found an association between exposure to PCBs and their effects on the immune system (Hertz-Picciotto et al., 2008). Epidemiologically, an increase in the rate of upper respiratory tract infections was demonstrated (Dallaire et al., 2004). At the pathophysiological level, changes in thymus development, changes in levels and differentiation of lymphocytes of the various subtypes, an increase in Immunoglobulin E (IgE) antibody levels in the umbilical cord, and a decrease in antibody production in response to childhood vaccines are described in association with PCBs exposure (Reichrtová et al., 1999; Weisglas-Kuperus et al., 2000; Belles-Isles et al., 2002; Heilmann et al., 2006). IgE antibody levels in the umbilical cord were found to be a predictor of atopic predisposition (Liptay et al., 1992). A recent study showed that serum aryl hydrocarbon receptor (AhR) bioactivities were increased with specific PCB congeners (Park et al., 2017). AhR activation was positively correlated with atopic dermatitis (Hidaka et al., 2017). It has been hypothesized that prenatal exposure to PCBs may be linked to atopic phenomena. Maternal PCBs levels were positively associated with upper respiratory tract in infections during the first year of life (Stølevik et al., 2013). Children exposed in-utero to persistent organic pollutants (POPs) such as organochlorine pesticides and PCBs have an increased risk of asthma, wheezing and eczema (Hansen et al., 2014; Mamane et al., 2015; Parker-Lalomio et al., 2017). There is a complex interaction between the environmental exposures acting during the early stages of development and genetic susceptibility that might contribute to asthma and allergy (Kahr et al., 2015; Morales and Duffy, 2019). Studies attempting to answer this question yielded conflicting results (Smit et al., 2015; Hansen et al., 2016; Parker-Lalomio et al., 2017).

There are prominent developmental events *in utero*, leading to critical windows for susceptibility to immunotoxic effects. Pregnant women and the fetus, infants, and children are most vulnerable to low-dosage environmental exposure. There is growing evidence of adverse effects of environmental exposure on reproduction, pre and post-natal development (Sharpe and Irvine, 2004; Di Renzo et al., 2015; Teysseire et al., 2019). As a lipophilic substance, PCBs can be transferred through the placenta and during breastfeeding to increase children’s body burden. Therefore, children are exposed to PCBs starting from conception, during pregnancy and lactation, and the exposure continues throughout lifetime (Sly and Flack, 2008).

This study aimed to examine the possible relationship between prenatal background exposure to PCBs and the prevalence of atopic phenomena in children from a birth cohort of Israeli mothers and children.

## Material and Methods

### Study Population

We used data collected from a birth cohort recruited at Shamir Medical Center (Assaf Harofe) and “Dana Dwek” Children’s Hospital, Tel Aviv Medical Center. Complete details of this birth cohort have been presented elsewhere (Sheinberg et al., 2020; Berlin et al., 2021).

Briefly, from January 2013 through April 2015, 263 mother-father-newborn triads were recruited. The women recruited for the study were asked to participate during attendance in the delivery room. Data on social and demographic characteristics and lifestyle variables from both the father and mother were obtained through a detailed questionnaire. Data on occupation, residential history, diet, hobbies, and detailed health history were also collected. At the delivery room, blood and urine samples were collected from mothers and fathers.

Birth weight, length, and head circumference were measured three times using standard research procedures. Birth weight was adjusted to gestational age at birth and infant’s sex and classified according to percentile values derived from the Israeli Perinatal survey (Dollberg et al., 2005).

All participants signed informed consent. The study was performed according to the Declaration of Helsinki, and the Institutional Review Board approved the protocol (The World Medical Association, 2018).

### Data Extraction

The database included 263 mother-newborn dyads. Due to budget limitations, we measured concentrations of 4 PCBs from maternal blood of 183 mothers who gave birth at Shamir Medical Center (Assaf Harofeh). On average, included and excluded dyads were similar in potential confounders such as birth weight, length of gestation, and maternal age (Sheinberg et al., 2020; Berlin et al., 2021).

Cases of women with twin pregnancies (*N* = 2), premature delivery (<37 weeks) (*N* = 8), and incomplete details (*N* = 3) were excluded.

We retrieved data on 170 dyads for which serum PCBs measurements were obtained. Data on maternal demographics, exposures, lifestyle, and labor were extracted as well as newborn anthropometrics and measurements.

Maternal laboratory test results obtained during delivery included 4 PCB congeners, total cholesterol, and triglycerides.

A follow-up questionnaire was sent out to participants between April 2019 and October 2019, when the children were 4–6 years old. Mothers were contacted to evaluate the prevalence of atopic phenomena among their offspring. The atopic symptoms were assessed using—the International Study of Asthma and Allergies in Childhood (“ISAAC”) questionnaire, (Asher et al., 1995), previously validated and implemented to assess the prevalence and severity of asthma and allergic reactions in children (Romano-Zelekha et al., 2007; Raherison et al., 2019; Jøhnk et al., 2020). The questionnaire includes modules to assess asthma, allergic rhinitis, and atopic dermatitis.

The outcomes of asthma, allergic rhinitis, and atopic dermatitis were classified as doctor-diagnosed or parent-reported. Parent-reported asthma symptoms were defined as children with one of the following symptoms: wheezing or shortness of breath in the past year or use of inhalations in the past year. Parent-reported symptoms of allergic rhinitis were defined as children with one of the following symptoms: chronic rhinitis occurring not as part of an acute infection in the last year or use of nasal sprays in the previous year. Parent-reported symptoms of atopic dermatitis were defined as children with one of the following symptoms: a persistent itchy rash in the past year or use of topical preparations in the past year.

The questionnaire was translated into Hebrew, and questions were added regarding general medical conditions and the presence of possible confounders such as smoking by one of the family members; child’s births order in the family; rural/urban living environment; pet in the family home; and atopy among nuclear family members.

### Sample Analysis

All samples were processed, aliquoted, and frozen at -80°C until analysis. PCBs were measured at the Centre de Toxicologie du Quebec. Congeners 118 (2,3′,4,4′,5-pentachlorobiphenyl), 138 (2,2′,3,4,4′,5′-hexachlorobiphenyl), 153 (2,2′,4,4′,5,5′-hexachlorobiphenyl), and 180 (2,2′,3,4,4′,5,5′-heptachlorobiphenyl) were measured. All measures were performed using GC/MS at INSPEQ, at the Arctic Monitoring and Assessment Programme Ring Test for Persistent Organic Pollutants in Human Serum (AMAP), organized and managed by the Centre de Toxicologie du Québec (CTQ).

The lower limit of quantification (LOQ) was 10 ng/L. For statistical analysis purposes, values below LOQ were assigned with the value of LOQ/√2. All the samples were above the LOQ for PCB 153. There were 11 samples with levels below the LOQ: PCB 118—six samples, PCB 118 + 138—one sample, PCB 118 + 180—one sample.

PCBs were measured in 200 randomly selected samples. Seventeen of the 200 samples were duplicates. The correlations between the duplicates were 0.996, 0.997, 1, and 0.997 for congeners 118, 138, 153, and 180, respectively. Where duplicate samples were measured, a mean of the two measures was taken.

PCBs were reported as the wet weight. We normalized the concentrations using the following equation, which adjusts chemical per gram of total lipids (lipid weight, ng chemical/g total lipid) (Schisterman et al., 2005).

Total lipids (TL) were estimated using the formula (Bernert et al., 2007; Bergonzi et al., 2009):

TL(g/L)=2.27×TC(g/L)+TG(g/L)+0.623

where TL—total lipids, TC—total cholesterol, TG—triglycerides.

Maternal total cholesterol and triglycerides were measured in the Shamir (Assaf Harofeh) biochemistry lab using standard methods. The enzymatic method was used to quantitatively determine cholesterol and triglycerides in human serum and plasma on Roche/Hitachi Cobas c systems.

### Statistical Analysis

Continuous variables are presented as mean and standard deviation (±SD) or median and interquartile range (IQR). Continuous variables were compared between groups using the Kruskal Wallis test or Mann-Whitney test. Categorical variables were compared using the Chi-square test, or Fisher’s exact test, as appropriate.

Spearman correlation coefficients were calculated to assess the correlation between continuous variables of the mother and the newborn and PCBs level variables.

Logistic regression models were constructed and OR and 95%CI were calculated. The models were adjusted for potential confounders: maternal age and family member with asthma/atopic condition. PCBs levels used were adjusted to serum lipids. In order to perform the regression analysis and to increase the sample size for each outcome, the studied outcomes (asthma, allergic rhinitis and atopic dermatitis) were combined into 3 groups—children with symptoms and/or with diagnosis.

All statistical tests were two-sided, and *p* < 0.05 was considered statistically significant. SPSS software (IBMS SPSS Statistics for Windows, Version 25, IBM Corp, Armonk, New York, United States) was used for all statistical analyses.

## Results

### Subjects’ Characteristics

**FIGURE 1:**
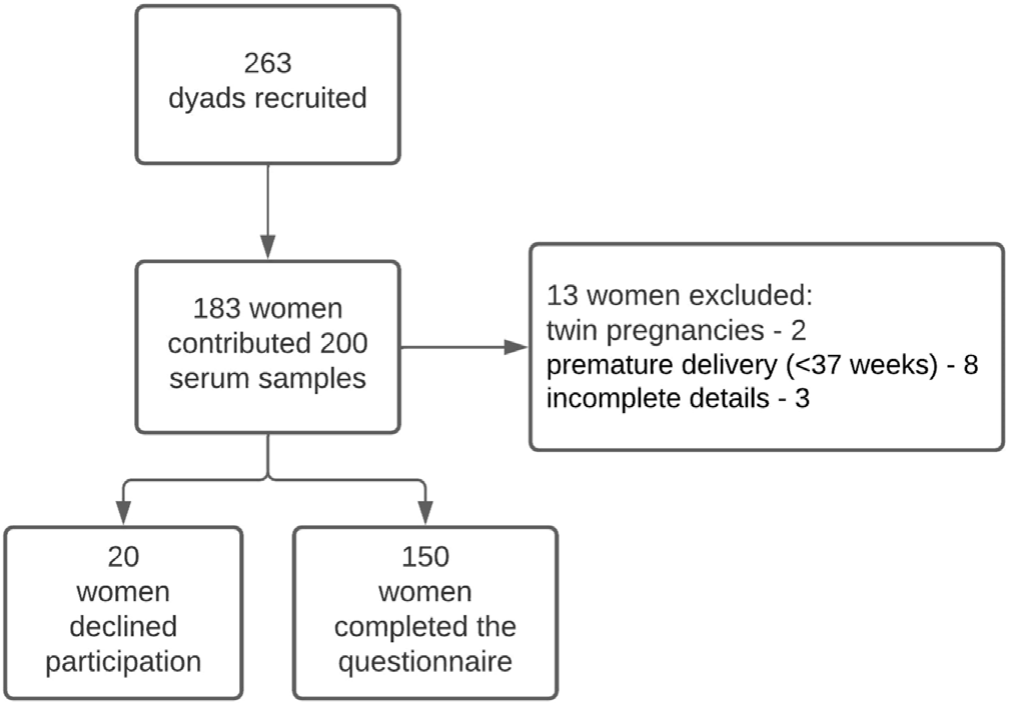
Flowchart of study population.

One hundred and fifty mothers-newborn dyads completed the questionnaire and were included in the final analysis, Figure 1 presents the flowchart of the study population. Maternal and newborn characteristics of those included in the final analysis and those who refused participation in the study are presented in Table 1. The only observed difference between the groups was that newborns included in the study had higher birth weight and birth percentile as compared to those who refused to answer the questionnaire, 3,200 (2,900–3,478) g vs. 2,875 (2,640–3,337) g, *p*-value = 0.03, birth percentile 53 (34–75) vs. 33 (12–70.75), *p*-value = 0.02.

**TABLE 1:** Selected maternal and child characteristics in the EHF-Assaf-Harofeh-Ichilov birth cohort, Israel, 2013–2015, comparing those included (*n* = 150) with those refused to answer the questionnaire (*n* = 20).

	Included *N* = 150	Refused participation *N* = 20	*p*-value
Maternal age at childbirth (years)	32 (30–36)	30.5 (29–35.5)	0.33
Maternal education (years)	16 (14–17)	16 (12.25–17)	0.58
Gestational age (weeks)	39 (38–40)	39 (38–40)	0.71
Birth weight (g)	3,200 (2,900–3,478)	2,875 (2,640–3,337)	**0.03**
Birth weight percentile	53 (34–75)	33 (12–70.75)	**0.02**
Child sex—male (N, %)	79 (52.7%)	8 (40%)	0.28

Values presented as median and IQR, unless otherwise specified.

**Bold**: *p*-value<0.05.

When comparing maternal, neonatal, and demographic characteristics between children with and without a diagnosis of studied outcomes, no statistically significant difference was found in most of the parameters, such as maternal age at childbirth, gestational age, birth weight percentile, current weight percentile, order of the child in the family, sex (male), smoker in the immediate family, rural living environment and presence of a household pet (Supplementary Tables S1–S6).

We found that 5% (*N* = 8) of the children were diagnosed with asthma. Parent-reported symptoms of asthma had 16% (*N* = 24) of children. Six children (4%) were diagnosed with allergic rhinitis, but for 21 children (14%), parents reported having allergic rhinitis symptoms. The prevalence of atopic dermatitis diagnosis was 13% (*N* = 19), and symptoms of atopic dermatitis were reported in 14 children (9%).

### Maternal Serum Polychlorinated Biphenyls Levels and Offsprings’ Allergic Outcomes

Statistical analysis was performed to examine the correlation between maternal PCB levels at birth and the other characteristics. Two associations were found to be statistically significant—maternal age and maternal education, the effect size was 0.45 (*p* < 0.01) and 0.33 (*p* < 0.01) for total PCBs, respectively (Table 2). Moderate-strong association was found between maternal age at birth and every studied congener—PCB 118–0.33, PCB 138—0.33, PCB 153—0.41, PCB 180—0.63, with *p*-value for all results <0.01. The association between the maternal education and studied PCBs was moderate—PCB 118—0.31, PCB 138—0.32, PCB 153—0.30, PCB 180—0.33, with *p*-value for all results <0.01 (Table 2).

**TABLE 2:** Spearman rank correlations of PCBs (lipid adj.) and maternal age and education in the EHF-Assaf-Harofeh-Ichilov birth cohort, Israel, 2013–2015 (*n* = 150).

	Maternal age	Maternal education
PCB 118 (ng/g lipids)	0.33	0.31
PCB 138 (ng/g lipids)	0.33	0.32
PCB 153 (ng/g lipids)	0.41	0.30
PCB 180 (ng/g lipids)	0.63	0.37
Sum PCBs (ng/g lipids)	0.45	0.33

*p*-value <0.01 (for all results presented).

We compared serum PCBs concentration (lipid-adjusted) in mothers of children with an allergic outcome to serum concentration in mothers without allergic outcome (Table 3). No significant differences were found in the median serum PCBs concentrations of 4 studied congeners or total PCBs for asthma, allergic rhinitis, atopic dermatitis diagnosis, or parent-reported symptoms.

**TABLE 3:** Maternal median (IQR) serum PCBs concentration (lipid adjusted) among offspring with or without allergic outcomes.

	—	Sum PCBs	P	PCB 118	P	PCB 138	P	PCB 153	P	PCB 180	P
Asthma diagnosed	with 8	11.71 (9.19; 20.57)	0.29	1.93 (1.74; 2.13)	0.12	2.95 (2.16; 4.36)	0.36	4.19 (3.22; 7.73)	0.3	3.26 (2.06; 6.65)	0.48
	w/o 142	15.03 (11.76; 19.99)		2.31 (1.85; 3.05)		3.36 (2.53; 4.81)		5.39 (4.10; 7.27)		3.72 (2.69; 5.39)	
Asthma symptoms	with 24	12.92 (10.01; 18.49)	0.10	2.07 (1.63; 2.73)	0.1	2.87 (2.16; 4.18)	0.13	4.47 (3.46; 6.84)	0.07	3.34 (2.25; 4.69)	0.16
	w/o 126	15.14 (12.02; 21.56)		2.31 (1.87; 3.17)		3.39 (2.61; 4.9)		5.48 (4.23; 7.73)		3.9 (2.71; 5.61)	
Allergic rhinitis diagnosed	with 6	15.18 (9.66; 31.03)	0.90	1.93 (1.39; 4.54)	0.42	3.9 (2.13; 7.11)	0.82	5.77 (3.59; 11.95)	0.79	3.7 (2.24; 8.16)	0.92
	w/o 144	14.69 (11.26; 19.64)		2.28 (1.82; 3.04)		3.31 (2.52; 4.63)		5.38 (3.9; 7.12)		3.69 (2.69; 5.38)	
Allergic rhinitis symptoms	with 21	14.48 (10.03; 20.56)	0.61	1.97 (1.73; 2.54)	0.15	3.22 (2.29; 4.68)	0.65	5.38 (3.59; 7.68)	0.68	3.26 (2.26; 5.13)	0.51
	w/o 129	14.75 (11.71; 20.29)		2.31 (1.84; 3.11)		3.34 (2.52; 4.84)		5.38 (4.04; 7.39)		3.75 (2.75; 5.42)	
Atopic dermatitis diagnosed	with 19	20.88 (12.59; 33.08)	0.19	2.52 (1.81; 4.67)	0.21	4.09 (2.25; 7.45)	0.15	7.73 (4.45; 12.25)	0.16	4.68 (2.48; 8.39)	0.30
	w/o 131	14.60 (11.15; 19.09)		2.24 (1.81; 2.94)		3.27 (2.52; 4.48)		5.21 (3.85; 6.90)		3.69 (2.69; 5.01)	
Atopic dermatitis symptoms	with 14	21.09 (12.46; 38.06)	0.14	2.39 (1.89; 4.77)	0.31	4.41 (2.75; 7.86)	0.14	7.79 (4.49; 14.37)	0.12	5.79 (2.64; 10.51)	0.08
	w/o 136	14.63 (10.97; 19.14)		2.25 (1.79; 2.97)		3.28 (2.51; 4.49)		5.38 (3.82; 6.93)		3.62 (2.68; 5.01)	

PCBs, polychlorinated biphenyls presented in (ng/g lipids).

IQR, interquartile range.

P—*p*-value.

w/o—without.

### The Association Between Maternal Serum Polychlorinated Biphenyls Levels and Asthma, Allergic Rhinitis and Atopic Dermatitis Diagnosis and/or Symptoms

We divided the serum PCBs concentration in tertiles, defined as concentrations in the lowest third, middle third, and highest third. Then compared the number of children with allergic conditions between first and second tertiles vs. the third tertile of maternal lipid-adjusted PCBs serum concentration (Table 4). No significant differences were found for asthma diagnosis or parent-reported symptoms. The same results were observed for allergic rhinitis diagnosis. A statistically significant difference was observed for PCB 118 in children with parent-reported allergic rhinitis when comparing the first and second tertile to the third tertile—18 vs. 6%, *p*-value = 0.05 (Table 4). A significantly higher percentage of children with atopic dermatitis diagnosis was observed in the third tertile of total PCBs—20.4 vs. 8.9% (in the first and second tertiles), *p*-value = 0.05. The same results were observed for the PCB 153. When comparing the parent-reported symptoms for atopic dermatitis–the same results were observed with a *p*-value toward significance. For total PCBs: 16.3 vs. 5.9% (third tertile vs. first and second tertiles), *p*-value = 0.07, the same results were obtained for PCB 153 and PCB 180 (Table 4).

**TABLE 4:** Comparison of number of children with allergic conditions, between first + second tertiles vs. third tertile of maternal lipid adjusted PCBs serum concentration.

	Sum PCBs			PCB 118			PCB 138			PCB 153			PCB 180		
	1 + 2	3d	P	1 + 2	3d	P	1 + 2	3d	P	1 + 2	3d	P	1 + 2	3d	P
Asthma diagnosed	5.9% (6)	4.1% (2)	1.0	7% (7)	2% (1)	0.27	6% (6)	4% (2)	0.72	5.9% (6)	4.1% (2)	1.0	5.9% (6)	4.1% (2)	1.0
Asthma symptoms	17.8% (18)	12.2% (6)	0.38	18% (18)	12% (6)	0.35	17% (17)	14% (7)	0.64	17.8% (18)	12.2% (6)	0.38	17.8% (18)	12.2% (6)	0.38
Allergic rhinitis diagnosed	4.0% (4)	4.1% (4)	1.0	5% (5)	2% (1)	0.66	3% (3)	6% (3)	0.4	4% (4)	4.1% (4)	1.0	4% (4)	4.1% (4)	1.0
Allergic rhinitis symptoms	13.9% (14)	14.3% (7)	0.94	18% (18)	6% (3)	**0.05**	14% (14)	14% (7)	1.0	12.9% (13)	16.3% (8)	0.57	13.9% (14)	14.3% (7)	0.94
Atopic dermatitis diagnosed	8.9% (9)	20.4% (10)	**0.05**	10% (10)	18% (9)	0.17	10% (10)	18% (9)	0.17	8.9% (9)	20.4% (10)	**0.05**	9.9% (10)	18.4% (9)	0.14
Atopic dermatitis symptoms	5.9% (6)	16.3% (8)	**0.07**	8% (8)	12% (6)	0.55	7% (7)	14% (7)	0.23	5.9% (6)	16.3% (8)	**0.07**	5.9% (6)	16.3% (8)	**0.07**

Values presented as % (N).

PCBs, polychlorinated biphenyls.

P—*p*-value.

Bold: *p*-value toward significance.

1 + 2 - first and second tertiles.

3d—third tertile.

The studied outcomes (asthma, allergic rhinitis and atopic dermatitis) were combined into 3 groups—children with symptoms and/or with diagnosis to increase the sample size. No association was found between exposure to Sum PCBs and the risk for asthma symptoms and/or diagnosis, crude: OR = 0.96, 95%CI: (0.91; 1.01) or adjusted to maternal age and family member with atopic condition: aOR = 0.94, 95%CI: (0.88; 0.99) (Table 5). No association was observed between each studied PCB congener and asthma symptoms or diagnosis. The same results were found also for other studied outcomes—allergic rhinitis and atopic dermatitis (Table 5).

**TABLE 5:** The association between PCBs (lipid-adjusted) serum levels and asthma, allergic rhinitis and atopic dermatitis diagnosis and/or symptoms.

	N	Sum PCBs		PCB 118		PCB 138		PCB 153		PCB 180	
		OR crude	aOR CI 95%	OR crude	aOR CI 95%	OR crude	aOR CI 95%	OR crude	aOR CI 95%	OR crude	aOR CI 95%
Asthma symptoms and/or diagnosis	26	0.96 (0.91; 1.01)	0.94 (0.88; 0.99)	0.74 (0.51; 1.08)	0.67 (0.45; 1.01)	0.86 (0.69; 1.07)	0.8 (0.63; 1.02)	0.91 (0.8; 1.04)	0.86 (0.74; 1.01)	0.88 (0.73; 1.06)	0.74 (0.57; 0.97)
Allergic rhinitis symptoms and/or diagnosis	22	0.99 (0.95; 1.03)	0.98 (0.94; 1.03)	0.91 (0.64; 1.21)	0.88 (0.65; 1.2)	0.96 (0.81; 1.13)	0.94 (0.78; 1.12)	0.98 (0.89; 1.07)	0.96 (0.87; 1.07)	0.94 (0.8; 1.1)	0.89 (0.72; 1.1)
Atopic dermatitis symptoms and/or diagnosis	21	1.02 (0.99; 1.04)	1.01 (0.98; 1.04)	1.16 (0.96; 1.4)	1.11 (0.9; 1.36)	1.07 (0.96; 1.2)	1.04 (0.91; 1.18)	1.03 (0.97; 1.09)	1.01 (0.94; 1.08)	1.07 (0.97; 1.18)	1.01 (0.9; 1.14)

PCBs, polychlorinated biphenyls presented in (ng/g lipids).

aOR, adjusted odd ratio for maternal age, family member with asthma/atopic condition.

CI 95% - 95% confidence interval.

## Discussion

In this prospective birth cohort study of 150 mothers-newborns dyads, we aimed to evaluate the possible associations between background exposure to PCB congeners (118, 138, 153, and 180) and children’s allergic conditions. We found that a significantly higher percentage of children with atopic dermatitis diagnosis or parent-reported symptoms of atopic dermatitis were detected in the third tertile of maternal serum levels of total PCBs, PCB 153 and PCB 180. There were no statistically significant associations between prenatal PCBs exposure and asthma or allergic rhinitis in the offspring aged 4–6 years. However, after performing the logistic regression and adjusting the analysis for maternal age and family member with atopic condition, no association was found for each of the atopic conditions—asthma, allergic rhinitis and atopic dermatitis.

The prevalence of symptoms of asthma, allergic rhinitis, and atopic dermatitis in the last 12 months in our study (16, 14, 9.3%, respectively) is comparable to those previously reported in an Israeli national study conducted using the same questionnaires from 2003 (13.8, 10.5, 8.7%) (Romano-Zelekha et al., 2007). The higher rates in our study could be attributed to the lower age in our study (4–6 vs. 12–13 years). Additionally, contrary to our study, the aforementioned study used self-report of diagnosis to define the disease and not self-report of symptoms. Data from other industrialized countries based on Phase 3 of ISSAC studies also indicate prevalence rates of allergic rhinitis ranging around 15% (Björkstén et al., 2008).

In our study, children with atopic dermatitis diagnosis or symptoms were located in higher tertile of the studied PCBs. There was no statistically significant difference in the median maternal serum levels of PCBs in children with or without diagnosis or parent-reported symptoms of atopic dermatitis. However, the median levels of total PCBs were still much higher in groups with diagnosis or symptoms of atopic dermatitis—20.88 (12.59; 33.08) ng/g lipids vs. 14.60 (11.15; 19.09) ng/g lipids for diagnosis and 21.09 (12.46; 38.06) ng/g lipids vs. 14.63 (10.97; 19.14) ng/g lipids for symptoms of atopic dermatitis. Aside from genetic factors, the recent dramatic increase in the prevalence of atopic dermatitis in low- and middle-income countries strongly suggests that environmental factors may play an essential role in the pathogenesis of atopic dermatitis (Narla and Silverberg, 2020). The role of maternal exposure to PCBs in atopic dermatitis is unclear.

A previous retrospective study showed that reported prenatal exposure to PCBs increased the odds for eczema/hay fever [OR 3.29 (1.54–7.04)] (Parker-Lalomio et al., 2017). In mice, maternal exposure to DEHP during neonatal periods was found to accelerate atopic dermatitis-like skin lesions related to mite allergen in male offspring, possibly via T helper 2 (TH2)-dominant responses (Yanagisawa et al., 2008). Another study did not find a correlation between levels of environmental chemical contaminants in maternal serum in pregnancy and childhood rates of eczema at 5–9 years (Hansen et al., 2016). In contrast, some studies showed prenatal PCB exposures were inversely associated with a history of atopic dermatitis (Grandjean et al., 2010; Ochiai et al., 2014). A cross-sectional study conducted amongst Japanese adults found exposure to certain PCBs (dioxins) associated with a reduced risk of atopic dermatitis (Nakamoto et al., 2013).

Similar to atopic dermatitis, studies assessing the role of maternal exposure to PCBs in wheezing/asthma and allergic rhinitis show conflicting results (Björkstén et al., 2008; Grandjean et al., 2010; Smit et al., 2015; Parker-Lalomio et al., 2017). It is possible that due to multiple confounders, different PCBs examined, different methods and timing of testing, and relatively small cohorts, results in various studies are conflicting.

Atopic dermatitis is considered the first manifestation of the atopic march, and therefore relatively young age of questioning in our study could be related to the underdiagnosis of children who will later present with other atopic phenomena (Yang et al., 2020).

The birth weight and birth percentile of the newborns included in the analysis were higher as compared to the refusal group (Table 1). This is a potentially selection bias. However, the birthweight was within the normal range in both groups (appropriate for gestational age). Furthermore, the refusal group included a small number of newborns (*N* = 20), as compared to the study group (*N* = 150).

Our study has several strengths. It is a prospective birth cohort study with a high response rate in the study’s second phase. Analysis based on patient-reported symptoms could very well be more representative of actual prevalence, as the underdiagnosis of atopic conditions is reported in resource-rich and developing countries (Brożek et al., 2013; Esteban et al., 2014; Jacobs et al., 2014; Krajewska-Wojtys et al., 2016; Aaron et al., 2018; Yang et al., 2019). PCBs 118, 138, 153, 180 are among the most frequently detected congeners in white adipose tissue (Müllerová and Kopecký, 2007), and those specific PCBs were tested in our cohort. As the information collected relies on self-reporting, it is subject to recall bias. To minimize this bias, we considered information from the last year before the data collection date to include patients in the atopic groups, which could have resulted in over including some children who had acute rather than chronic conditions in the atopic groups. On the other hand, children with acute conditions, common in younger children, whose symptoms later did not persist were not included in the atopic groups, strengthening the analysis.

## Conclusion

This study demonstrated that children with atopic dermatitis diagnosis or symptoms are located at higher tertile of maternal PCBs level. No similar relationship was demonstrated for asthma or allergic rhinitis. The logistic regression adjusted for maternal age and family member with atopic condition, found no association for each of the atopic conditions—asthma, allergic rhinitis and atopic dermatitis. Additional multi-participant studies, with longer, spanning into later pediatric age follow up are needed to examine the possible effects of other environmental pollutants on the prevalence of atopic phenomena. Our research joins a series of previous studies that attempt to shed light on environmental exposures in utero as influencing factors for atopic conditions in children; as in previous studies, the results reflect the complexity of the pathophysiology of these phenomena.

## Data Availability

The raw data supporting the conclusion of this article will be made available by the authors, without undue reservation.
