# Supplementary material for: Maternal Exposure to Polychlorinated Biphenyls and Asthma, Allergic Rhinitis and Atopic Dermatitis in the Offspring: The Environmental Health Fund Birth Cohort
Source: Front Pharmacol. 2022 Apr 6;13:802974. doi: 10.3389/fphar.2022.802974 (PMC9019472; doi:10.3389/fphar.2022.802974)
Supplement: Supplementary file 1 [file DataSheet1.docx]

**Supplemental Tables**

**Table 1**

**Comparison of maternal, neonatal and demographic characteristics between children with and without diagnosis of asthma**

| P value | Children without diagnosis of asthma, n=142 | Children with diagnosis of asthma, n=8 |  |
| --- | --- | --- | --- |
| **0.02** | 16 (14-17) | 18.5 (16.25-19.75) | Mean maternal education (years) |
| 0.90 | 32 (30-36) | 30 (29-40) | Mean maternal age at childbirth (years) |
| 0.24 | 39 (38-40) | 38 (38-39) | Mean gestational age (weeks) |
| 0.33 | 53 (36-75) | 32 (10-85) | Mean birth weight percentile |
| 0.76 | 50 (25-75) | 37.7 (4.1-92.2) | Mean current weight percentile |
| 0.15 | 2 (1-3) | 1 (1-2) | Order of child in the family |
| 0.28 | 73 (51.4%) | 6 (75%) | Sex (male) |
| 0.71 | 45 (31.6%) | 3 (37.5%) | Smoker in the immediate family |
| 0.99< | 20 (14%) | 1 (12.5%) | Rural living environment |
| 0.11 | 43 (30%) | 5 (62.5%) | Presence of household pet |
| **0.03** | 46 (32.4%) | 7 (87.5%) | Family member asthma/atopic conditions |

Values presented as median and IQR, unless otherwise specified.

**Bold**: P-value<0.05.

**Table 2**

**Comparison of maternal, neonatal and demographic characteristics between children with and without parent-reported symptoms of asthma**

| P value | Children without parent-reported symptoms of asthma, n=126 | Children with parent-reported symptoms of asthma,  n=24 |  |
| --- | --- | --- | --- |
| 0.64 | 16 (14-17) | 16 (12.75-17.75) | Mean maternal education (years) |
| 0.08 | 31 (29.5-36) | 35 (30-38) | Mean maternal age at childbirth (years) |
| 0.44 | 39 (38-40) | 39 (38-39.75) | Mean gestational age (weeks) |
| 0.67 | 52 (32-75) | 56.50 (33-79.75) | Mean birth weight percentile |
| 0.68 | 50 (25-75) | 32.50 (8.75-83.75) | Mean current weight percentile |
| 0.25 | 2 (1-3) | 3 (1-3) | Order of child in the family |
| 0.29 | 64 (50.8%) | 15 (62.5%) | Sex (male) |
| 0.89 | 40 (32%) | 8 (33.3%) | Smoker in the immediate family |
| 0.20 | 20 (15.9%) | 1 (4.2%) | Rural living environment |
| 0.40 | 42 (33.6%) | 6 (25%) | Presence of household pet |
| **0.04** | 40 (31.7%) | 13 (54.2%) | Family member asthma/atopic conditions |

Values presented as median and IQR, unless otherwise specified.

**Bold**: P-value<0.05.

**Table 3**

**Comparison of maternal, neonatal and demographic characteristics between children with and without diagnosis of allergic rhinitis**

| P value | Children without diagnosis of allergic rhinitis, n=144 | Children with diagnosis of allergic rhinitis, n=6 |  |
| --- | --- | --- | --- |
| **0.02** | 16 (14-17) | 17 (16-19.25) | Mean maternal education (years) |
| 0.90 | 32 (30-36) | 32 (29.75-40.75) | Mean maternal age at childbirth (years) |
| 0.24 | 39 (38-40) | 39 (38-39.25) | Mean gestational age (weeks) |
| 0.33 | 55 (36.5-75.5) | 16 (9.5-25.5) | Mean birth weight percentile |
| 0.76 | 50 (25-75) | 3.25 (1.25-38.75) | Mean current weight percentile |
| 0.15 | 2 (1-3) | 2 (1-3.75) | Order of child in the family |
| 0.28 | 75 (52%) | 4 (66%) | Sex (male) |
| 0.71 | 47 (32%) | 1 (16%) | Smoker in the immediate family |
| 0.99< | 20 (13.8%) | 1 (16%) | Rural living environment |
| 0.11 | 46 (32%) | 2 (33%) | Presence of household pet |
| 0.19 | 49 (34.0%) | 4 (66.7%) | Family member asthma/atopic conditions |

Values presented as median and IQR, unless otherwise specified.

**Bold**: P-value<0.05.

**Table 4**

**Comparison of maternal, neonatal and demographic characteristics between children with and without parent-reported symptoms of allergic rhinitis**

| P value | Children without symptoms allergic rhinitis, n=129 | Children with symptoms allergic rhinitis, n=21 |  |
| --- | --- | --- | --- |
| 0.63 | 16 (14-17) | 16 (13.5-18) | Mean maternal education (years) |
| 0.34 | 31 (29.25-36) | 32 (30-39) | Mean maternal age at childbirth (years) |
| 0.16 | 39 (38-40) | 39 (39-40) | Mean gestational age (weeks) |
| 0.82 | 52.5 (35.5-75) | 56 (24.5-72.5) | Mean birth weight percentile |
| **0.01** | 50 (25-75) | 22.5 (4.1-50) | Mean current weight percentile |
| 0.49 | 2 (1-3) | 2.5 (1.25-3) | Order of child in the family |
| 0.81 | 67 (51.9%) | 12 (57%) | Sex (male) |
| 0.13 | 38 (29.7%) | 10 (47.6%) | Smoker in the immediate family |
| 0.73 | 19 (14.7%) | 2 (9.5%) | Rural living environment |
| 0.53 | 40 (31.3%) | 8 (38.1%) | Presence of household pet |
| 0.20 | 43 (33.3%) | 10 (47.6%) | Family member asthma/atopic conditions |

Values presented as median and IQR, unless otherwise specified.

**Bold**: P-value<0.05.

**Table 5**

**Comparison of maternal, neonatal and demographic characteristics between children with and without diagnosis of atopic dermatitis**

| P value | Children without diagnosis of atopic dermatitis, n=131 | Children with diagnosis of atopic dermatitis, n=19 |  |
| --- | --- | --- | --- |
| 0.71 | 16 (14-17) | 15.5 (12.75-17.25) | Mean maternal education (years) |
| 0.33 | 32 (29.75-36) | 31 (29.75-40.25) | Mean maternal age at childbirth (years) |
| **0.03** | 39 (38-40) | 38 (37.75-39) | Mean gestational age (weeks) |
| 0.86 | 52.5 (35-5-73.5) | 54.5 (25-75-88.25) | Mean birth weight percentile |
| 0.33 | 50 (25-75) | 62.5 (28.75-90) | Mean current weight percentile |
| 0.08 | 2 (1-3) | 2 (1-2) | Order of child in the family |
| 0.43 | 71 (54%) | 8 (42%) | Sex (male) |
| 0.08 | 39 (29%) | 9 (47%) | Smoker in the immediate family |
| 0.72 | 18 (13.7%) | 3 (0.33%) | Rural living environment |
| 0.24 | 40 (30.8) | 8 (42%) | Presence of household pet |
| **0.03** | 42 (32.1%) | 11 (57.9%) | Family member asthma/atopic conditions |

Values presented as median and IQR, unless otherwise specified.

**Bold**: P-value<0.05.

**Table 6**

**Comparison of maternal, neonatal and demographic characteristics between children with and without parent-reported symptoms of atopic dermatitis**

| P value | Children without symptoms of atopic dermatitis, n=136 | Children with symptoms of atopic dermatitis, n=14 |  |
| --- | --- | --- | --- |
| 0.73 | (14-17) 16 | (12-18.25) 15 | Mean maternal education (years) |
| 0.10 | (29-36) 32 | (30-41.5) 35 | Mean maternal age at childbirth (years) |
| 0.15 | (38-40) 39 | 38.5 (38-39) | Mean gestational age (weeks) |
| 0.60 | (36-75) 53 | 48.5 (17.5-62.5) | Mean birth weight percentile |
| 0.28 | (25-75) 50 | (15-57.5) 25 | Mean current weight percentile |
| 0.64 | (1-3) 2 | 2 (1-4) | Order of child in the family |
| 0.57 | (53.6%) 73 | 6 (42%) | Sex (male) |
| 0.14 | (30.4%)41 | 7 (50%) | Smoker in the immediate family |
| >0.99 | (14%) 19 | (14.3%)2 | Rural living environment |
| >0.99 | (32.6%) 44 | 4 (28.6%) | Presence of household pet |
| 0.56 | 47 (34.6%) | 6 (42.9%) | Family member asthma/atopic conditions |

Values presented as median and IQR, unless otherwise specified.

**Bold**: P-value<0.05.
